# Peritoneal Dialysis in Pediatric Postoperative Cardiac Surgical Patients

**DOI:** 10.5005/jp-journals-10071-23221

**Published:** 2019-08

**Authors:** Manoj Kumar Sahu, Bipin C, Yatin Arora, Sarvesh Pal Singh, V Devagouru, P Rajshekar, Shiv Kumar Chaudhary

**Affiliations:** 1,2,4 Intensive Care for CTVS, Department of CTVS, CN Centre, All India Institute of Medical Sciences, New Delhi, India; 3,5–7 Department of Cardiothoracic and Vascular Surgery, CN Centre, All India Institute of Medical Sciences, New Delhi, India

**Keywords:** Cardiopulmonary bypass, Pediatric cardiac surgery, Peritoneal dialysis, Postoperative period

## Abstract

**Background:**

We determined the prevalence of acute kidney injury requiring peritoneal dialysis (PD), the factors associated with early PD initiation, prolonged PD and mortality among pediatric postoperative cardiac surgical patients.

**Materials and Methods:**

The hospital records of 23 children, aged 12 years or younger, who had undergone cardiac surgery and required PD subsequently, during a 1-year period were reviewed. Demographic data, intraoperative variables, and postoperative complications were compared between survivors and nonsurvivors of PD, between the short and long duration PD groups, and between the early and late PD initiation groups.

**Results:**

Six hundred and eight pediatric patients who underwent open heart surgery were enrolled in this study. 23 (3.78%) of them required PD. When compared with survivors (*n* = 11), non survivors (*n* =12) were more likely to have a higher serum procalcitonin (*p* = 0.01), higher serum potassium on day 2 (*p* = 0.001), day 3 (*p* = 0.04), day of termination of PD (*p* = 0.001) and a lower urine output on day 3 of PD (*p* = 0.03). Prolonged PD was associated with time of PD initiation (*p* = 0.01), a higher postoperative serum creatinine on day 3 (*p* = 0.01) of PD initiation as well on the day of PD termination (*p* = 0.01) and the final outcome in terms of survival (*p* = 0.02). Factors significantly associated with an early PD initiation were CPB time (*p* = 0.04), sepsis (*p* = 0.02) and shorter PD duration (*p* = 0.003).

**Conclusion:**

PD is very useful mode of renal replacement therapy among pediatric postoperative cardiac surgical patients. The intraoperative and postoperative variables have important association with the time of PD initiation, PD duration and patient survival.

**How to cite this article:**

Sahu MK, Bipin C, Arora Y, Singh SP, Devagouru V, Rajshekar P, *et al.* Peritoneal Dialysis in Pediatric Postoperative Cardiac Surgical Patients. Indian J Crit Care Med 2019;23(8):371–375.

## INTRODUCTION

Acute kidney injury (AKI) is a well-known complication which faces treatment challenges after pediatric cardiac surgery.^1–3^ Risk factors of AKI and the need for renal replacement therapy include young age, complex cardiac lesions, long cardiopulmonary bypass time, and a low cardiac output state after surgery.^4–6^ Although the choice of renal replacement therapy is controversial,^7,8^ Peritoneal dialysis (PD) has been shown to be useful because of the ease of application, effectiveness in fluid removal and avoidance of additional vascular access and anticoagulation.^1–3^,^6–8^ Many studies have documented the importance of young age as a determinant of AKI and PD after pediatric cardiac surgery.^3–5^ A retrospective analysis was performed in 23 young children aged 12 years or younger undergoing open heart surgery for congenital heart diseases. We determined the prevalence of AKI requiring PD. We also determined the risk factors association with prolonged PD, early PD initiation and mortality.

## MATERIALS AND METHODS

Between July 2017 and August 2018, 726 children were admitted in our cardiac surgical intensive care unit (CSICU) after undergoing cardiac surgery for different congenital cardiac anomalies. Premature babies, children above 12 years, those who underwent closed heart operations and emergency surgeries were excluded. A total of 118 children were excluded from the study. Remaining 608 children were included. Fifty-seven children developed renal impairment (AKI) and 34 of them recovered with hemodynamic optimization and diuretic therapy. Remaining 23 children required PD in the postoperative period. The hospital records of these 23 patients, aged 12 years or younger were reviewed. The following data were collected: demographic information, complete cardiac diagnosis, surgical procedures performed, complexity of surgery (classified into 4 risk categories according to Aristotle Comprehensive Complexity score, with category 4 procedures having the highest risk), cardiopulmonary bypass (CPB) and aortic cross clamp (ACC) duration, postoperative complications including mortality, indications of PD, timing of PD initiation, duration of PD, urine output (UO), serum potassium (S. K^+^) and creatinine (S. cr) levels on days 1 to 3 and day of PD termination and PD related complications.

Prolonged PD was defined as the PD duration exceeding 5.5 days and short PD as PD duration of 5.5 days or less in this study (5.5 days was the mean PD duration of the study population). Late PD was defined as the PD initiation 3.5 days after the CSICU admission and early PD as PD initiation 3.5 days before CSICU admission after open heart surgery (3.5 days was the mean time period for PD initiation after ICU admission in this study population). This study was started after obtaining approval from the Institute's Ethics committee, but the consent from individual patients was waived off considering the retrospective nature of the study.

Peritoneal dialysis is the first treatment of choice for AKI in postoperative pediatric cardiac surgical patients at our institution. AKI was defined as decreased urine output < 0.5 mL/kg/ h for more than 6 hours or serum creatinine rising more than 0.3mg/dl during last 24 hours, despite aggressive diuretic therapy and optimization of inotropic support, or a combination of both (AKIN criteria).^9^ We in our institute do not have a protocol of initiating intraoperative PD prophylactically. Postoperatively PD is initiated whenever it is indicated. Indications for PD included AKI as defined above, severe fluid overload due to third spacing caused by CPB induced severe inflammation, low cardiac output(LCO) situation leading to severe metabolic acidosis(pH <7.2, base deficit > −10) and hyperkalemia (K^+^ exceeding 5 mEq/L). All the patients who developed metabolic acidosis and/or hyperkalemia, were treated medically before initiating peritoneal dialysis. Indications for stopping PD included return of sufficient urine output, normalization of serum electrolytes and acid-base status.

The PD catheter (Romsons scientific and surgical industries Pvt Ltd, Agra, UP, India) was inserted percutaneously through the infraumbilical approach in the CSICU under complete aseptic measures. Initiation of PD was in accordance with the preceding indications. The PD catheter was connected to a closed system for peritoneal drainage. The dialysate solution used were standard commercial preparations (Shree Krishna Keshav Lab Ltd, Ahmedabad, Gujarat, India) with a dextrose concentration of 1.7%. Heparin (500 units/L of dialysate) was added. PD was started with a dwell volume of 10–20mL/kg, a fill time of 10 minutes, a dwell time of 30 minutes, a drainage time of 20 minutes and at one hourly cycles. According to the targeted fluid balance, acidosis, hyperkalemia and hemodynamics, the dwell volume, time and PD cycles were changed subsequently if needed. The dialysate bags were changed every 24 hours. Serum albumin was regularly monitored, and intravenous albumin (20%) infusions were given as necessary.

Data were expressed as median with inter quartile range as the total number of study participants were only 23. Categorical variables were analyzed using Chi-square test or Fischer test, while non-categorical variables were analysed using Man-whitney test or t-test as appropriate. A *p* value of less than 0.05 was considered statistically significant. All statistical analyses were performed using STATA/SE 14.0 software.

## RESULTS

### Demography

In the present study, 23 patients among the 608 young children who underwent open heart surgery, experienced AKI or excessive fluid load or acidosis and required PD. Thus the prevalence of PD was 3.78%. The surgical procedures performed and their risk categories are shown in Table 1. The mean age of these 23 patients at surgery was 22.12 months (range 0.2–108 months), and their mean body weight was 8.42 kg (range 1.8–25.0 kg). The mean time interval between surgery and institution of PD was 3.5 days (range 0–15 days), whereas the mean duration of PD was 5.5 days (range, 2–10 days).

### Factors Associated with Survival Following PD among Pediatric Cardiac Surgery Patients (Table 2)

There were a total of 12 (52%) deaths among the 23 who received PD. Factors significantly associated with survival after starting PD were serum procalcitonin (*p* = 0.01), serum potassium on day 2 (*p* = 0.005), day 3 (*p* = 0.04), day of stopping the PD(*p*=0.001) and urine output on day 3 of PD (*p* = 0.03).

**Table 1:** Cardiac surgery of 23 pediatric patients who required PD postoperatively

*Sl. No*	*Cardiac surgery*	*No. of patients (n = 23)*	*Age in months*	*Risk (surgical complexity score)*	*No of survivors (n = 11) [Survivor = s/nonsurvivor = n]*
1	ASD closure + sliding Tracheoplasty	1	2	2	1[s]
2	ASO	1	3	4	0[n]
3	ASO+ASD closure	1	2.5	4	1[s]
4	ASO+ ECMO	1	1	4	0[n]
5	ASO+VSD closure	1	5	4	1[s]
6	AVSD repair+ TAP	3	48/48/84	3	2[n/s/s]
7	AVSD repair	1	24	2	0[n]
8	DSO	1	108	4	0[n]
9	ICR	2	36/12	2	1[n/s]
10	ICR+TAP	3	12/1/48	3	2[n/s/s]
11	ICR + TAP + LPA plasty	1	12	4	0[n]
12	REV procedure	1	12	4	0[n]
13	TAPVC repair	2	0.3/0.2	2/3	1[n/s]
14	Truncus repair	1	24	2	0[n]
15	Truncus repair + IAA repair	1	1	4	0[n]
16	VSD closure (severe PAH)	1	1	2	1[s]
17	VSD closure + TAP	1	24	2	1[s]

For the purpose of statistical analysis, scores 1 and 2 were clubbed together as low risk and scores more than two as high risk category

**Table 2:** Comparison of clinical variables between survivors and nonsurvivors following peritoneal dialysis

*SI No*	*Variable*	*Survivor (n = 11) Median (p25, p75)*	*Nonsurvivor (n = 12) Median (p25, p75)*	*p value*
1	Age in months	5(1,24)	12(2,42)	0.52
2	Sex (no of male : female)	7:4	8:4	0.88
3	Weight in kg	7.6(3.2,10)	9.25(5.7,11)	0.25
4	Complexity of surgery (low : high score)	6:5	5:7	0.54
5	Preoperative hospitalization (days)	3.5 (2,11)	6 (1,11)	0.94
6	CPB time in minutes	120 (114,136)	138 (96,150)	0.70
7	ACC time in minutes	75 (66,85)	69 (64,108)	0.85
8	Sternum (open : closed)	4:7	4:8	0.88
9	Time of PD initiation in days	2 (1,6)	3 (1,4)	0.88
10	Days PD ran	5 (4,6)	7.5 (4,9)	0.15
11	Procalcitonin in ng/ml	10 (2,18)	22 (15,150)	0.01
12	Sepsis (microbial culture = Sterile: Positive)	8:3	10:2	0.54
13	S.K+ day1 in mEq/L	4.1 (3.9,4.9)	4.15 (3.9,4.8)	0.97
14	S.K+ day2 in mEq/L	4.1 (3.6,4.5)	4.95 (4.65,5.6)	0.001
15	S.K+ day3 in mEq/L	3.9 (3.6,4.2)	5.1 (4.6,5.5)	0.04
16	S.K+ day of PD termination in mEq/L	3.87 (3.5,4.1)	4.95 (4.4,5.25)	0.001
17	S.cr. on day1 PD (mg/dL)	0.6 (0.5,1.3)	0.7 (0.4,0.9)	0.94
18	S.cr. on day2 PD (mg/dL)	0.9 (0.5,1.5)	1.1 (0.9,1.3)	0.43
19	S.cr. on day3 PD (mg/dL)	1 (0.6,1.3)	1.3 (1.15,1.55)	0.25
20	S.cr day of PD termination (mg/dL)	1 (0.3,2.2)	1.65 (1.55,1.95)	0.32
21	UO day 1 of PD in mL/kg/hr	1.157(0.23,2.9)	1.3 (0.61,2.9)	0.60
22	UO day 2 of PD in mL/kg/hr	1.56 (0.16,2.6)	0.405 (0.055,1.605)	0.31
23	UO day 3 of PD in mL/kg/hr	2.2 (0,3.64)	0 (0,0.245)	0.03
24	UO day of PD termination in mL/kg/hr	2.5 (1,4.1)	0.0915 (0.0175,0.21)	0.18

### Factors Associated with Longer PD duration among Pediatric Cardiac Surgery Patients (Table 3)

Factors significantly associated with a longer duration of PD were time of PD initiation (*p*=0.01), a higher postoperative serum creatinine on day 3 of PD (*p*=0.01), on the day of PD termination (*p*=0.01). Short duration PD was associated with decreased mortality during the postoperative period (*p*=0.02). In our study the odds ratio between the two groups of short duration and prolonged PD considering the complexity of surgery was 5.3 (95% CI = 0.8911 to 31.9191, *p* = 0.05).

### Factors Associated with Early PD Initiation among Pediatric Cardiac Surgery Patients (Table 4)

Factors significantly associated with an early PD initiation were CPB time (*p* = 0.04), culture positive sepsis (*p* = 0.02) and short PD duration (*p* = 0.003). And based on observations in Tables 3 and 4, it was found that early PD initiation was associated with lesser number of days of PD.

### Complications

PD and procedure related complications like peritonitis, or bleeding or bowel injury were not found in this subset of patients. Three children had mechanical problems of PD catheter misplacement/ malfunction needing repositioning /change.

## DISCUSSION

PD is a dependable, safe and effective method for treating AKI in young children in the postoperative period after open heart surgery. AKI in this subset of patients mostly related to the complex congenital pathology requiring longer extracorporeal circulation time for repair of the defects, the cyanosis, CPB induced inflammation and sepsis.^4–6^

The reported prevalence of AKI requiring PD in children after cardiac surgery with CPB range from 1.6 to 9%.^1–5^, ^10^ In our study the prevalence of AKI requiring PD was 3.78%, similar to the previous studies. The mortality rate of our patients undergoing PD was relatively high at 52%, even though it matches with previously described studies with rates of 27 to 79%.^1,4,7,11,12^ The high death rate in our cohort of patients was probably because of longer CPB, LCO and sepsis.

**Table 3:** Comparison of clinical variables between short PD and prolonged PD

*SI No*	*Variable*	*Short PD (n = 12) Median (p25, p75)*	*Long PD (n = 11) Median (p25, p75)*	*p value*
1	Age in months	12 (2.25,42)	5 (1,24)	0.42
2	Sex (no of male : female)	8:4	7:4	0.88
3	Weight in kg	8.25(5.4,10.25)	7.8(3.6,10)	0.59
4	Complexity of surgery (low : high score)	8:4	3:8	0.05
5	Preoperative hospitalization (days)	4(1,6)	10(1,11)	0.30
6	CPB time in minutes	117.5(105,137)	140(96,148)	0.75
7	ACC time in minutes	73.5(63,85.5)	66(66,96)	0.97
8	Open sternum: closed sternum	3:9	5:6	0.30
9	Time of PD initiation in days	1(1,3)	4(3,7)	0.01
10	No of survivors: nonsurvivors	8:4	3:6	0.02
11	Procalcitonin in ng/ml	12.5(2,20)	22(15,150)	0.06
12	Microbial culture (Sterile: Positive)	11:1	7:4	0.10
13	S.cr. on day 1 PD (mg/dL)	0.6(0.4,0.8)	0.7(0.5,1.3)	0.52
14	S.cr. on day 2 PD (mg/dL)	0.9(0.5,1.25)	1.2(0.9,1.5)	0.12
15	S.cr. on day 3 PD (mg/dL)	1(0.65,1.4)	1.5(1.3,1.9)	0.01
16	S.cr. on day of PD termination (mg/dL)	1.1(0.4,1.7)	1.8(1.6,2.2)	0.01

**Table 4:** Comparison of clinical variables between early PD and late PD

*SI No*	*Variable*	*Early PD (n = 15) Median (p25, p75)*	*Late PD (n = 8) Median (p25,p75)*	*p value*
1	Age in months	12 (1,36)	4 (1.5,36)	0.62
2	Sex (male : female)	10:5	5:3	0.84
3	Weight in kg	8 (3.6,10)	7.05 (3.05,11)	0.87
4	Complexity of surgery (low : high score)	7:8	4:4	0.88
5	Preoperative hospitalization(days)	3 (1,6)	10 (4.5,11)	0.09
6	CPB time in minutes	136 (114,146)	96(40,136)	0.04
7	ACC time in minutes	76 (66,96)	66(48,66)	0.07
8	Open sternum : closed sternum	5:10	3:5	0.84
9	No of survivors: nonsurvivors	7:8	4:4	0.88
10	Days PD ran	5(3,6)	8.5(6,9.5)	0.003
11	Procalcitonin in ng/mL	15(8,22)	19(8.5,125)	0.53
12	Microbial culture (Sterile: Positive)	14:1	4:4	0.02
13	S.cr. on day 1 PD (mg/dL)	0.6(0.3,0.9)	0.6(0.5,1.3)	0.64
14	S.cr. on day 2 PD (mg/dL)	0.9(0.5,1.3)	1.05(0.8,1.45)	0.53
15	S.cr. on day 3 PD (mg/dL)	1.3(0.7,1.6)	1.3(1.15,1.7)	0.36
16	S.cr day of PD termination (mg/dL)	1.5(0.5,1.8)	1.8(1.65,2.15)	0.07

In our study, factors significantly associated with survival following PD were serum procalcitonin levels, serum potassium on day 2, day 3, day of PD termination and urine output on day 3 of PD. However Guiffre et al compared clinical variables between survivors and nonsurvivors after PD and did not find any pre, intra or postoperative variables as risk factors.^13^ On the other hand, Chan et al.^14^ found that associated syndromes (Down's, DiGeorge's), requirement of preoperative ventilation, a higher postoperative ventilatory settings and LCOS were significant risk factors. As per Chien et al, risk factors included CPB & ACC time, pH, bicarbonate and base deficit before beginning PD, intervals between acute renal failure (ARF) and PD, PD and peak creatinine, PD and urine output on recovery, duration of inotropic agents, time of mechanical ventilation and duration of hyperglycemia.^15^ We in our study could not demonstrate any direct association between the preoperative hospital admission (p >0.05), delayed sternal closure (p >0.05) and the duration of PD and survival. One child with AVSD and PS was a case of Down's syndrome who did not survive, but we did not study on this aspect to comment on syndromes and their association with AKI.

We further determined factors associated with a longer duration of PD and those were the time of PD initiation, a higher postoperative serum creatinine on day 3 of PD, the day of PD termination. Also short duration PD had a significant survival advantage in the postoperative period. According to Chan et al, risk factors identified were younger age, higher preoperative serum creatinine, postoperative LCOS, pulmonary hypertensive crisis and a higher oxygen requirement.^14^ Chien et al. demonstrated that postoperative LCOS, prolonged inotropic therapy, postoperative ventilator settings, sepsis, disseminated intravascular coagulopathy, and multiple organ failure were the risk factors.^15^ In our study the odds ratio between the two groups of short duration and prolonged PD considering the complexity of surgery showed that children with higher surgical complexity score required longer duration of PD. Similar findings have been reported in the past by Dittrich et al. as AKI is often related to cyanotic heart disease, complex congenital cardiac anomalies and their operation, longer CPB time.^16^

The optimal timing for PD intervention is still controversial. Many authors have suggested that early intervention with PD is needed as soon as ARF occurs.^2,11,17^ Chien et al. from their study found that, the time to initiate PD in survivors was significantly earlier than that in nonsurvivors.^15^ We in our study demonstrated that early PD initiation was associated with lesser number of days of PD. The CPB time, culture positive sepsis and PD duration were the significant factors found to be associated with an early initiaton of PD in the present study. However we did not find any statistically significant survival benefit with early PD initiation.

Dittrich et al.^18^ in their study showed that PD can be performed safely in smaller children without affecting hemodynamics negatively, we too did not come across any major problems in the performance of PD. Though the serum creatinine decreased very slowly, those were normalized in all the surviving children and this was in agreement with other studies.^3,10^

### Study Limitations

Retrospective nature of this study was a drawback. Enrollment of small number of patients requiring PD in our study caused a low statistical power and we could not apply multivariate analysis to assess risk factors for prolonged PD and mortality. Some studies have highlighted the beneficial effects of PD on hemodynamics and cardiovascular function,^1,18^ which could have been assessed in a prospective manner. Hence more prospectively designed studies with larger number of study participants are needed to investigate the risk factors of prolonged PD and mortality in pediatric cardiac surgical patients with AKI.

## CONCLUSION

PD is very effective in achieving fluid, electrolyte and acid-base balance without causing significant hemodynamic compromise with minimal or no complications. Sepsis biomarker positivity (high PCT values) seem to be one of the major associated factors of postoperative mortality following PD. Short duration PD was associated with decreased mortality during the postoperative period. Early PD initiation seemed to have advantages in terms of reducing the total duration and subsequent morbidity.
